# Effect of boron deficiency on anatomical structure and chemical composition of petioles and photosynthesis of leaves in cotton (*Gossypium hirsutum* L.)

**DOI:** 10.1038/s41598-017-04655-z

**Published:** 2017-06-30

**Authors:** Mingfeng Li, Zhuqing Zhao, Zhihua Zhang, Wei Zhang, Jun Zhou, Fangsen Xu, Xinwei Liu

**Affiliations:** 1 Microelement Research Center, Huazhong Agricultural University, Wuhan, 430070 China; 2 Hubei Provincial Engineering Laboratory for New-Type Fertilizer, Wuhan, 430070 China; 3 College of Resources and Environment, Huazhong Agricultural University No. 1, Shizishan Street, Hongshan District, Wuhan, Hubei Province 430070 P. R. China

## Abstract

The main symptom of boron (B) deficiency in cotton is the formation of brown rings on leaf petioles. The objective of the present study was to determine the changes in the anatomical structure and chemical composition of petioles and photosynthesis of leaves in cotton under B deficiency. Compared to the control, B deficiency treatment resulted in large increases in the number of petioles with brown rings per plant (160.0%) and the number of rings on the petiole per functional leaf (711.1%) in cotton seedlings. The relative absorbance intensity in the fingerprint region of polysaccharide structure was decreased in petiole rings under B deficiency, while lignin contents were increased. Cotton plants mitigated the impairment of transport function in cotton petioles by increasing the areas of vascular bundles, phloem, xylem, and phloem fiber. Moreover, the stomatal conductance, photosynthetic rate, and transpiration rate in leaves were significantly decreased under B deficiency, thus impeding photosynthesis in cotton plants. Therefore, B deficiency reduces transport function in petioles and photosynthesis in leaves, and leads to the formation of noticeable brown rings on petioles of cotton seedlings.

## Introduction

Boron (B) is an essential nutrient element for plant growth and development^1^. Deficiency of B has been found in many regions of the world, such as Australia, New Zealand, Africa, Spain, the United States, Brazil, and China, where it seriously affects the development of local agriculture^2–4^. In China, the Yangtze River Basin, the Yellow River Basin, and the Northwest Inland are among the major cotton (*Gossypium hirsutum* L.) growing areas. In these areas, soil B is considered to be the most easily deficient trace element to cotton and therefore severely limits the growth and development of cotton plants. An investigation showed that soil B concentration was substantially low in cotton fields in Tianmen, Hubei Province, China; however, most local farmers applied no B fertilizer or only used compound fertilizers containing small amounts of B, resulting in significant symptoms of B deficiency in cotton, such as brown rings on leaf petioles and buds without blooming^5^.

Boron not only affects the formation and development of reproductive organs of plants^6–8^, but also plays an essential role in the vegetative growth of plants^9–12^. This element influences the transport and metabolism of photosynthetic products in plants^13–16^, and also participates in the structural composition of cell walls and membranes^17, 18^. It indirectly affects the metabolism of proteins and nucleic acids^18, 19^, and also mediates the levels of hormones and phenolic substances in the plant body^14, 20, 21^. The trace element B is difficult to move in plants and it is mainly transported in the xylem through the root pressure generated by transpiration or in the phloem through other mechanisms; the mobility of B in phloem is extremely low^22, 23^, resulting in a low degree of reutilization of B in plants. A transient deficiency of B can lead to irreversible damage to cotton plants and thus seriously affect cotton yield^24, 25^.

Previous research of B nutrition has mainly focused on the changes in plant roots, leaves, and pollens. For example, B deficiency inhibits root elongation^26, 27^, affects leaf growth and development^28–30^, and prevents pollen tube elongation^31^. An important reason is that the root and leaf are vital organs of plants to acquire nutrients, whereas the pollen is an important reproductive organ to obtain yield. The main symptom of B deficiency in cotton is the formation of brown rings on leaf petioles, accompanied by a series of physiological and ecological changes^5^. The petiole acts as an important support organ of cotton and transports nutrients, water, organics, and hormones through petiole vascular bundles to ensure the growth and development of cotton. However, little research has investigated the changes in petiole vascular bundles.

A few studies have investigated leaf vascular bundles under B deficiency and found that B deficiency affected the growth and led to the proliferation of vascular tissue in plants^32^. Additionally, B deficiency increased the cross-sectional area of spruce needles^33, 34^ and resulted in the proliferation of vascular bundles and lignin in leaf veins of sweet orange^35^. The formation of brown rings on leaf petioles is an important sign of potential B deficiency in cotton. However, the chemical composition of brown rings on cotton petioles has rarely been reported. The aim of the present study was to determine the changes in the anatomical structure and chemical composition of petioles and photosynthetic parameters of leaves in cotton under B deficiency.

## Results

### Changes in plant height and shoot biomass of cotton under B deficiency

**Figure 1:**
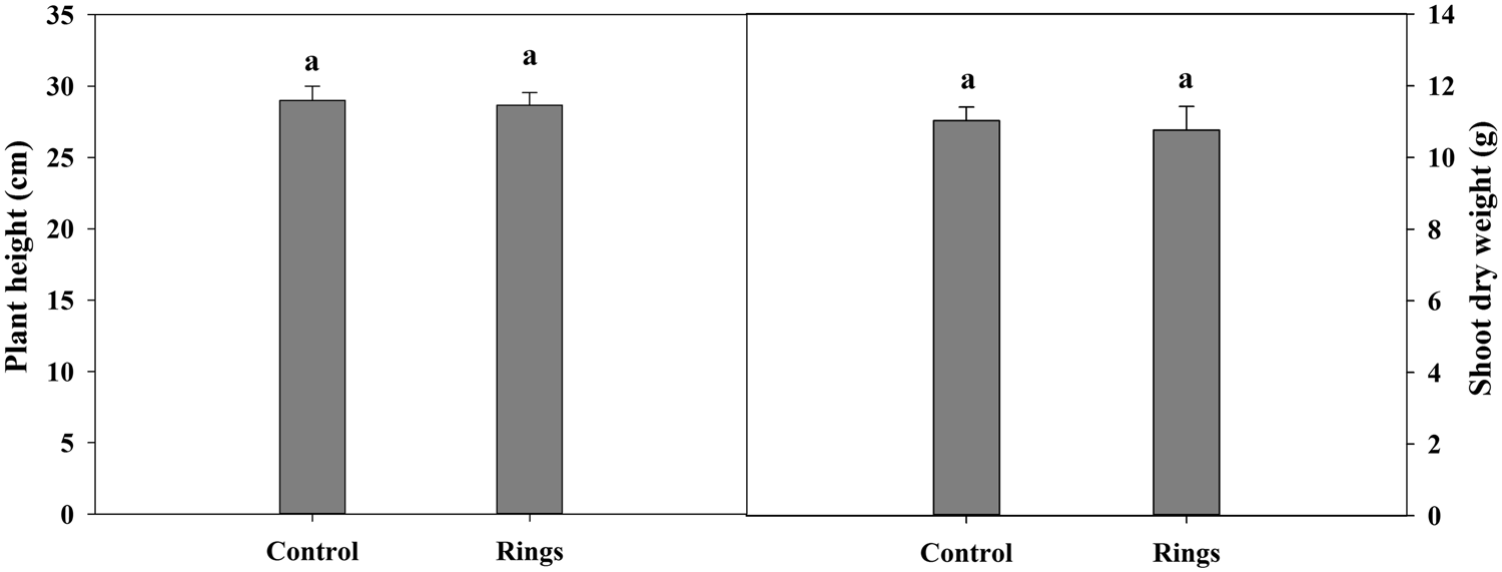
Effect of boron deficiency on plant height and shoot biomass of cotton. The data of six replicates are expressed as the mean + SE.

Plant height and shoot biomass were slightly, but not significantly, decreased after the formation of brown rings on cotton petioles under B deficiency compared to the controls (Fig. 1). Plant height declined from 29.00 to 28.67 cm and shoot biomass dropped by 0.26 g plant^−1^, indicating that B deficiency had little effect on the growth of cotton seedlings.

### Changes in the formation of brown rings on cotton petioles under B deficiency

**Figure 2:**
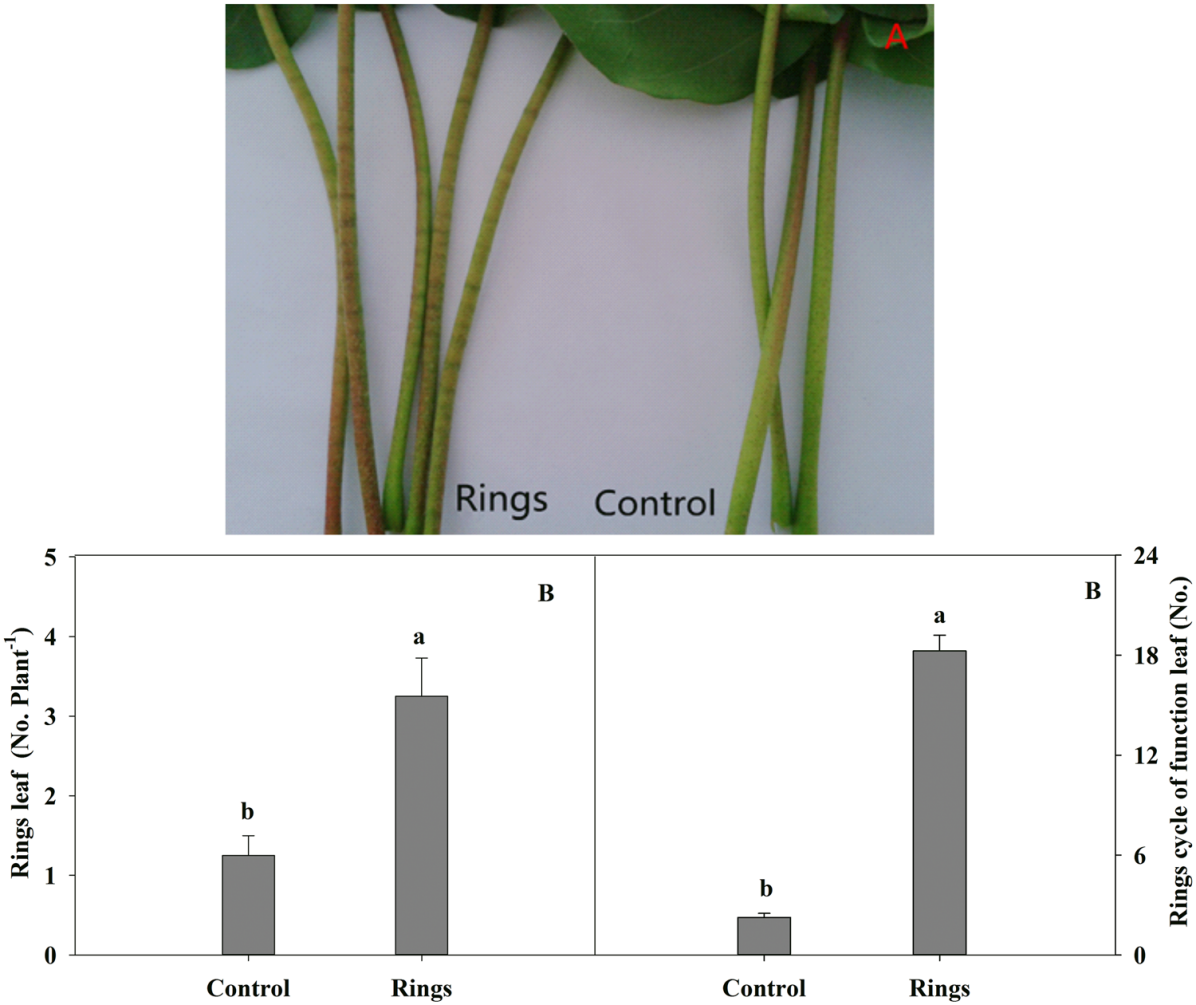
Effect of boron deficiency on the formation (**A**) and number (**B**) of brown rings on cotton petioles. The data of six replicates are expressed as the mean + SE.

Distinct brown rings were formed on petioles of cotton seedlings under B deficiency (Fig. 2A). The B deficiency treatment significantly increased the number of petioles with brown rings per plant and the number of rings on the petiole per functional leaf (Fig. 2B). Under B deficiency, these two parameters were increased by 160.0% and 711.1%, respectively, compared to the controls. This indicates that the level of ring formation on leaf petioles of cotton seedlings was significantly associated with B.

### Changes in FTIR spectra and XRD patterns of cotton petioles under B deficiency

**Figure 3:**
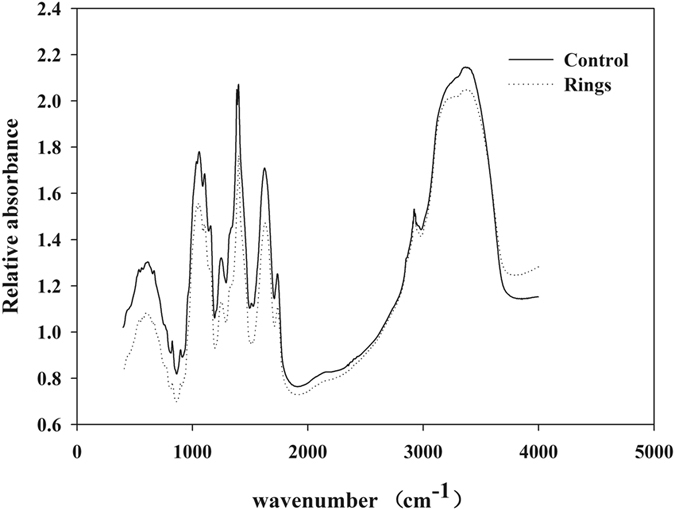
Changes in infrared absorption spectra of cotton petioles after formation of brown rings under boron deficiency compared with normal petioles (control).

FTIR spectra showed that both the position and relative absorbance of the absorption peak were changed to a certain degree in petiole rings compared to the control petioles (Fig. 3). This indicates that the chemical composition of cotton petioles changed after the formation of brown rings. In the wavelength region over the range of 900–1200 cm^−1^, known as the fingerprint region of polysaccharide structure^36^, the petiole rings showed a characteristic absorption peak at about 1149 cm^−1^, with the relative absorbance of 1.27; the control petioles showed a slightly higher absorption peak at about 1157 cm^−1^, with the relative absorbance of 1.46 (Fig. 3). In the petiole rings compared to the control petioles, the position of the absorption peak shifted towards low frequency by ~8 cm^−1^, while the relative absorbance of the absorption peak declined by ~0.19. These changes demonstrate that major changes occurred in the polysaccharide structure and content of petiole rings.

Additionally, a distinct absorption peak was found at around 1517 cm^−1^ (Fig. 3), which is characteristic of the benzene ring in phenols; the relative absorbance was markedly lower in the petiole rings than in the control petioles. The absorption bands at about 1550–1650 cm^−1^ were attributed to the amide group in cell wall proteins^37^. The characteristic absorption peaks at about 2930 and 2858 cm^−1^ were attributed to reversed stretching vibrations of -CH_2_ mainly from wax, protein, and pectin, among various tissue components in the cells^37^; the relative absorbance was markedly decreased in the petiole rings compared to the control petioles. The characteristic absorption peak at about 3400 cm^−1^ was attributed to OH stretching vibrations of carbohydrates, mainly from hydrogen bonding^38^; the relative absorbance was also significantly decreased in the petiole rings relative to the control petioles.

**Figure 4:**
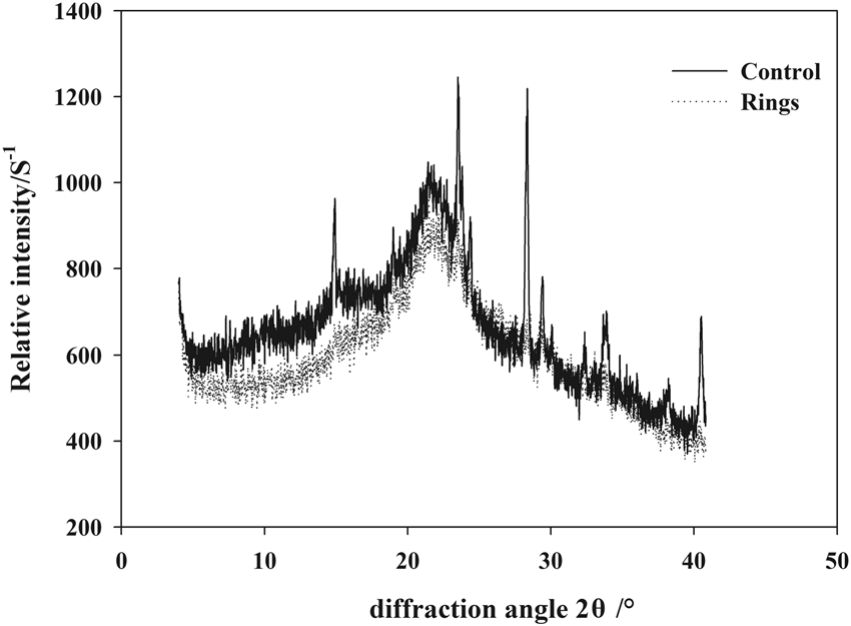
Changes in X-ray diffraction pattern of cotton petioles after formation of brown rings under boron deficiency compared with normal petioles (control).

The diffraction peak at near 2θ = 18° indicates the scattering intensity of the amorphous background diffraction. There were no significant differences in the amorphous background diffraction between the petiole rings and control petioles at 2θ = 18° (Fig. 4). Meanwhile, distinct diffraction peak appeared in the control petioles, whereas the diffraction peak intensity under B deficiency was markedly decreased and even mostly disappeared. These results indicate that B deficiency led to reduced cellulose crystallinity in cotton petioles.

### Changes in anatomical structure of cotton petioles under B deficiency

**Figure 5:**
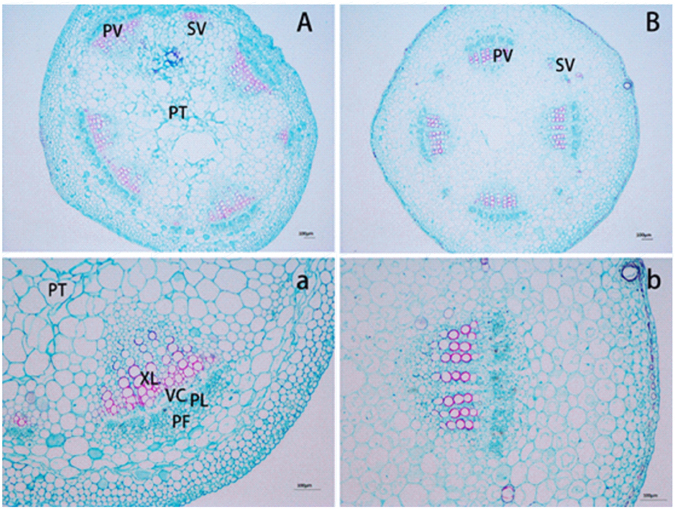
Microphotographs showing the changes in anatomical structure of cotton petioles after formation of brown rings under boron deficiency compared with normal petioles (control) (**A**) petiole ring; (**B**) normal petiole; (**a**) vascular cambium in petiole ring; and (**b**) vascular cambium in normal petiole. (PL) Phloem; (PF) Phloem fiber; (VB) Vascular bundle; (XL) Xylem; (PT) Pith; (PV) primary vascular bundles; (SV) secondary vascular bundles.

The effect of boron deficiency on the petiole of cotton was mainly demonstrated on the changes of vascular cells and pith cells. Microscopic observations (Fig. 5) showed that in the control petioles, primary and secondary vascular bundles were uniformly arranged and separated by parenchyma cells, while those in the petiole rings were interconnected, with vascular bundle cells expanded and deformed. In addition, in the control petioles, pith cells were mostly hexagonal or pentagonal and neatly arranged, while in the petiole ring, the cells were squeezed with each other and seriously deformed.

The petioles with brown rings were thicker and more brittle compared to the controls. Table 1 shows that the areas of vascular bundles, xylem, and phloem of petiole rings were significantly increased, by 15.8%, 14.8%, and 23.3%, respectively, compared to the controls. Both xylem vessel diameter and single vessel area of petiole rings were significantly decreased, by 11.1% and 26.9%, respectively (Table 1). The lignin contents in the petiole rings were increased by 50.4%, compared to the control petioles (Table 1).

**Table 1:** Changes in the composition of cotton petioles after formation of brown rings under boron (B) deficiency compared with normal petioles (control).

Treatment	Cross-sectional area of petiole (mm^2^)	Area of vascular bundles (mm^2^)	Area of xylem (mm^2^)	Area of Phloem (mm^2^)	Area of Phloem fiber (mm^2^)	Catheter diameter (μm)	Area of Catheter (mm^2^)	Lignin (%)
Control	4.73 ± 0.04a	0.95 ± 0.02b	0.27 ± 0.01b	0.30 ± 0.01b	0.16 ± 0.01a	33.20 ± 0.26a	940.66 ± 8.91a	8.04 ± 0.44b
Rings	4.85 ± 0.11a	1.10 ± 0.03a	0.31 ± 0.02a	0.37 ± 0.01a	0.17 ± 0.01a	29.53 ± 0.54b	687.3 ± 17.69b	12.09 ± 0.42a

Values are the mean ± SE of six replicates. Different lowercase letters in a column indicate statistically significant difference at *p* < 0.05.

### Changes in B concentrations and forms of cotton leaves under B deficiency

**Figure 6:**
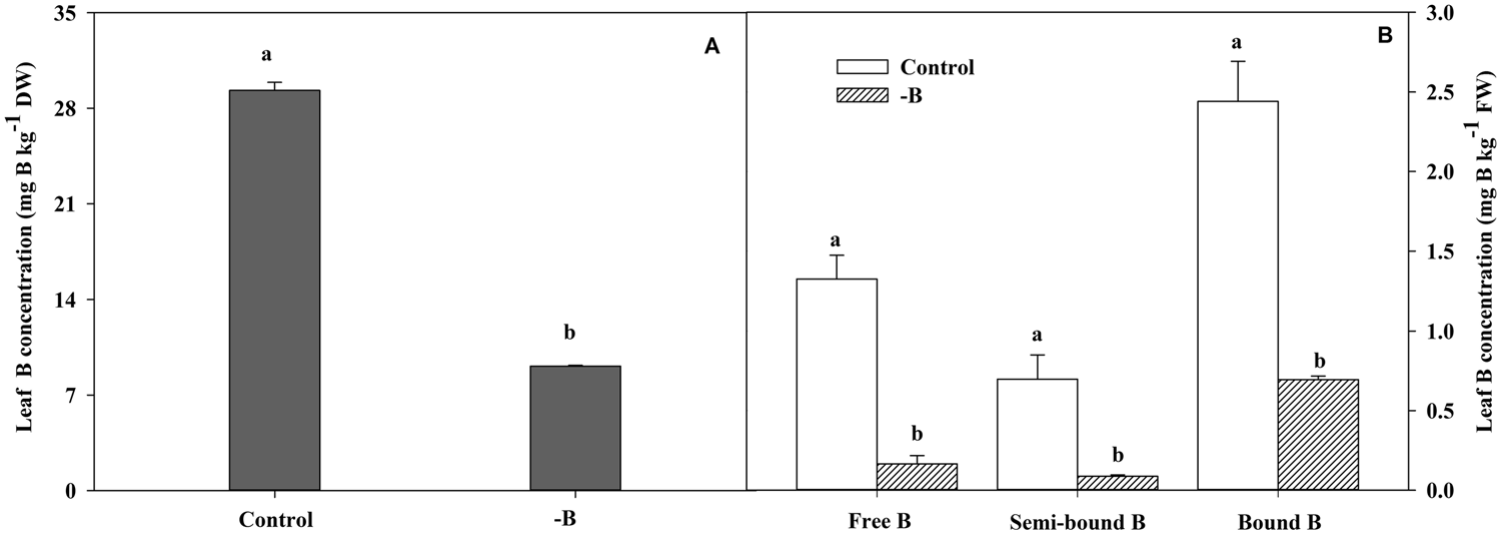
Changes in total boron concentration (**A**) and concentration of different forms (**B**) of cotton leaves after formation of brown rings on the petiole under boron deficiency compared with normal leaves (control). Total (**B**) was extracted from dry sieved functional leaves with 1 M HCl. Different forms of (**B**) were extracted from fresh functional leaves following a three-step procedure. The data of six replicates are expressed as the mean + SE.

The total B concentration of cotton leaves with brown rings on the petiole was significantly decreased, by 68.6%, compared to the control leaves (Fig. 6A), indicating that the total B of cotton leaves was significantly decreased under B deficiency. As shown in Fig. 6B, the B was mainly present in the bound form in cotton leaves. The concentration of different forms of B in cotton leaves was decreased after the formation of rings on the petiole. There were 87.2%, 87.1%, and 71.3% significant decreases in free B, semi-bound B, and bound B concentrations, respectively. This result indicates that the concentration of different forms of B in cotton leaves was affected under B deficiency, with the most significant effect on free B.

### Changes in chlorophyll content and photosynthetic parameters of cotton leaves under B deficiency

The area of functional leaves with brown rings on the petiole was significantly decreased, by 5.9%, while the relative chlorophyll content (represented by the measured SPAD value) was slightly, but not significantly, decreased by 3.7%, compared to the control leaves. Photosynthetic rate, transpiration rate, and stomatal conductance were significantly decreased, by 25.1%, 28.2%, and 28.1%, respectively, in cotton leaves after the formation of brown rings on the petiole (Table 2). These results indicate that the reduction in leaf area, transpiration rate and stomatal conductance is mainly responsible for the lower photosynthetic rate in cotton plants under potential B deficiency conditions.

**Table 2:** Changes in chlorophyll content and photosynthetic parameters of cotton leaves after formation of brown rings under boron (B) deficiency compared with normal petioles (control).

Treatment	Functional leaf area (cm^2^)	Chlorophyll content (SPAD value)	Photosynthetic rate (µmol m^−2^ s^−1^)	Transpiration rate (mmol m^−2^ s^−1^)	Stomatal conductance (mol H_2_O m^−2^ s^−1^)
Control	93.18 ± 1.69a	39.75 ± 0.77a	20.07 ± 0.55a	7.65 ± 0.50a	0.32 ± 0.02a
B deficiency	87.65 ± 3.32b	38.28 ± 0.93a	15.04 ± 1.46b	5.49 ± 0.39b	0.23 ± 0.03b

Values are the mean ± SE of six replicates. Different lowercase letters in a column indicate statistically significant difference at *p* < 0.05.

## Discussion

In this study, the B concentration in the functional leaves of cotton without B application (B deficiency treatment) was 9.12 mg kg^−1^ (Fig. 6A). Cotton is a crop with a high requirement for B, and the B concentration of less than 20 mg kg^−1^ in cotton leaves indicates B deficiency in cotton^39^. Therefore, the level of B supply in our experimental soil was insufficient to meet the growth and development requirement for cotton. The B exists mainly in three forms (free, semi-bound, and bound) in cotton plants, with most of B present in the cell walls^40^. Our results showed that the concentration of different forms of B in the functional leaves of cotton was markedly decreased after the formation of brown rings on leaf petioles, with the decrease in free B concentration up to 87.2% compared to the control (Fig. 6B). This is mainly because the available forms of B, including free and semi-bound B, could enter the cell walls to mitigate the damage caused by B deficiency^41^.

In the present study, both plant height and shoot biomass were slightly, but not significantly, decreased under B deficiency. The most distinct symptom of B deficiency in cotton lies in the formation of brown rings on leaf petioles^5^. It is known that the use efficiency of B depends largely on the translocation of B from the xylem to the phloem and its redistribution to new organs^42, 43^. In this study, we found that vascular bundles were most sensitive to boron deficiency, vascular bundles gradually proliferated and were even interconnected with each other. The main function of plant vascular bundles is the transmission of water, inorganic salts and organic matter, and the role of supporting plants^44^. The conduits of the xylem are mainly involved in carrying water, dissolving inorganic matter, and providing mechanical support for the plant. The screen and the companion cells of phloem mainly transport photosynthetic products. The direct product of photosynthesis is transported from the leaves through the stem vascular bundle to the other parts. Therefore, the vascular organization is an important transmission organization, and the number and size of vascular bundles directly affect the vascular transport of material transport capacity and mechanical support strength. Vascular cambium increased in thickness, and cambium division into phloem cells increased in cotton plants, leading to increases of 15.8%, 14.8%, and 23.3%, respectively, in the areas of vascular bundles, xylem, and phloem in the brown rings on leaf petioles, over those of the control. We speculate that, under B deficiency, cotton plants could regulate a series of stress responses to mitigate the damage. The increases in the area of vascular bundles would contribute to the translocation of nutrients in cotton plants. Moreover, the lignin contents were increased in the brown rings on leaf petioles.

Deficiency of B has been shown to induce changes in the metabolism of carbohydrates, relevant enzymes, and phenols in plants^14, 20, 21^. In the present study, FTIR spectra (Fig. 3) were acquired to demonstrate the main chemical components in plant cells. The relative absorbance intensity in the fingerprint region of polysaccharide structure, i.e., 900–1200 cm^−1^, was reduced in the petioles with brown rings under B deficiency, compared to the controls. It can be inferred that, under B deficiency, more B is transformed to semi-bound B by binding to monosaccharide or polysaccharide, and then adapt to the B-deficient conditions by binding to cell walls. Studies have shown that B deficiency results in an increase in carbohydrates of plant leaves^45^. In this study, we found that carbohydrates in the petioles were declined relative to the control, which may be attributed to a decline in the transport function of petiole and the accumulation of carbohydrates in the leaves.

Cellulose crystallinity refers to the percentage of crystalline region in the overall cellulose, and generally, the higher the cellulose crystallinity, the stronger the physical properties (e.g., tensile strength) of the plant^46^. The spectral absorption in this region is associated with not only polysaccharide structure but also crystal structure^47^. The XRD patterns showed that the cellulose crystallinity of cotton petioles was decreased under B deficiency, resulting in fragile petioles with lower mechanical strength.

In FTIR analysis, the infrared absorption peak corresponds to the functional group characteristics of specific chemical component^48^. FTIR spectra showed that the intensity of absorption peak characteristic of the amide group in proteins was markedly decreased in the brown rings formed on cotton petioles. This indicates that B deficiency could result in lower glycoprotein content in cotton petioles, possibly because the B deficiency condition affects nitrogen metabolism in plants^49^ and thereby prevents protein synthesis in cotton petioles. Hu^50^ assessed the relationship between leaf B content and cell wall pectin content in 14 plant species and found a significantly positive correlation between the two parameters, with more than 70% of B in pumpkin and tobacco leaves bound to pectin in cell walls. In the present study, the absorption peak observed at about 1736 cm^−1^ was attributed to stretching vibrations of the ester group in esterified pectin^37^. The intensity of this absorption peak was relatively decreased in the brown rings on cotton petioles compared with the control petioles, indicating a certain relationship between cell wall pectin content in cotton petioles and plant B content.

Wojcik *et al*.^51^ reported that both root activity and photosynthetic rate were lowered in plants under B deficiency, leading to growth inhibition. Vascular bundles are the main channel of material transport in plants. In plants, B deficiency may affect photosynthesis by affecting leaf area and altering blade components^29, 52^. In the present study, the area of functional leaves after the formation of brown rings on the petiole was decreased, by 5.9%, compared to the control leaves, but no significant changes were found in the chlorophyll content of functional leaves, suggesting that the reduction in leaf area for photosynthesis is mainly responsible for the lower photosynthetic rate in cotton plants under potential B deficiency conditions. Additionally, it has also been reported that boron deficiency reduces photosynthetic efficiency by changing stomatal density and stomatal conductance to decrease the conductivity of CO_2_^53^. In the present study, we also found the significant decrease of stomatal conductance, photosynthetic rate, and transpiration rate in the functional leaves of cotton after the formation of brown rings on the petiole. We found that vascular bundles were most sensitive to boron deficiency. The vascular bundle of petiole to the destruction by boron deficiency. The deformation of the phloem sieve affects the transport of carbohydrates, and the deformation of the xylem ducts affects the transport of water and nutrients, resulting in reduced stomatal conductance and transpiration, which indirectly affects the transport of carbohydrates. The data showed that the leaves affected by boron deficiency had lower photosynthetic activity and the carbohydrate accumulation of petioles decreased. Studies have shown that boron deficiency leads to an increase in carbohydrate accumulation in leaves^45^. Therefore, the destruction of petiole vessels impairs the transport of photosynthetic products of the leaves to the other parts. This demonstrates that B plays a critical role in the photosynthesis and transport of nutrients in cotton plants.

## Conclusions

For the formation of brown rings on petioles of cotton in soils under potential B deficiency, vascular bundle vessels were initially compressed and destructed, which subsequently declined the transport function of cotton petioles, impaired the transport function of cotton petioles, impeded the transport of photosynthetic products of the leaves to the other parts through the petioles, and finally reduced the contents of polysaccharides, proteins, pectin, and carbohydrates in cotton petioles. In order to alleviate the blockage of transport caused by B deficiency, cotton plants responded with an increase in the areas of vascular bundles, phloem and xylem in the petiole rings, leading to the formation of brown rings on petioles of cotton. Therefore, B deficiency may inhibit the growth and development of cotton by impairing the transport function of petioles, preventing the transport of nutrients, reducing the photosynthetic capacity of plants, and decreasing the transport of photosynthetic products.

## Materials and Methods

### Soil and plant sample description

The experiments were conducted in a potting site in Huazhong Agricultural University (Wuhan, Hubei Province, China). A calcareous alluvial soil was collected from a cotton field in the county of Tianmen, Hubei Province. One week before sowing, 12 kg of 2 mm sieved soil was mixed with an indicated amount of basal fertilizer and filled into buckets of diameter 25 cm and height 30 cm. The basic soil physical and chemical properties were: pH 7.65 (soil water ratio of 1:2.5), organic matter 14.52 g kg^−1^, available N 62.19 mg kg^−1^, Olsen-P 3.29 mg kg^−1^, available K 61.09 mg kg^−1^, available B 0.10 mg kg^−1^. Seeds of the cotton (*Gossypium hirsutum* L.) cultivar ‘Tongza 411F_1_’ were purchased from Hubeiseed (Wuhan, Hubei Province, China).

### Pot experimental design

Cotton seeds were soaked in 40 °C water for 2 h and then placed on a sterile sponge filled with ultrapure water. The seeds were covered with sterile gauze for germination. Twenty-four hours later, seeds with similar plumpness and sprouting were selected and transplanted into pots. The pots were covered with a transparent film until the emergence of seedlings, and the seedlings were thinned to one plant per pot. Two treatments were performed, with six replicates and two B levels, 0 and 2 mg kg^−1^. Petioles with brown rings (B deficiency treatment) were obtained without B application, and normal petioles (control treatment) were obtained with B application at the rate of 2 mg kg^−1^. Other fertilizers were applied by mixing with the soil: N, 0.6 g kg^−1^; K_2_O, 0.32 g kg^−1^; and P_2_O_5_, 0.16 g kg^−1^.

### Sample preparation

Functional leaves (the fourth expanded leaf from the top) and their petioles were sampled 45 d after sowing. The leaf samples were immediately washed and then divided into two portions. One portion was frozen with liquid nitrogen and then stored at −80 °C for determination of different forms of B. The other portion was deactivated at 105 °C for 30 min and dried at 65 °C to constant weight, then ground and sieved for further use. Brown rings were cut down from the petioles of functional leaves in the B deficiency treatment. The rings, as well as the petioles of functional leaves in the control treatment, were deactivated at 105 °C for 30 min and dried at 65 °C to constant weight, then ground and sieved for further use.

### Determination of morphological parameters

Forty-five days after sowing, plant height was measured from the base of the stem to the terminal bud using a steel ruler. Aboveground stems and leaves were washed, oven dried, and weighed to determine shoot biomass. The number of leaves with rings on the petiole per plant was counted for each treatment. The number of rings on the petiole per leaf was counted for functional leaves of each plant. Leaf area was measured by the gravimetric method. Briefly, an A4 paper was weighed using a 0.0001 g analytical electronic balance and the area of the paper was also measured. A functional leaf was fixed on the A4 paper and the leaf shape was defined, clipped off, and weighed using the electronic balance. The accurate area of the functional leaf was calculated according to the area-to-weight ratio.

### Determination of chemical composition and anatomical structure

The test sample consisted of petiole rings. Due to the small number of rings collected from the functional leaf blade petiole, it was difficult to extract the cell wall. Additionally, due to serious petiole lignification, we directly studied the properties of the cell wall and protoplasts of the rings. We used the cytoplasm and cell wall of the petiole as a whole to discuss the changes in the normal petiole and the petiole rings.

X-ray diffraction (XRD): Powder samples of sieved petiole rings and control petioles were placed on the sample rack and automatically scanned. The scan was performed using an X-ray tube with a copper (Cu) target, and a nickel sheet to eliminate Cu-Ka radiation. Other parameters were as follows: tube current, 40 mA; tube voltage, 40 kV; scanning range, 5–45°; scanning step, 0.02; scanning rate, 4 °C min^−1^. The XRD measurement was performed using a JDX-10P3A X-ray powder diffractometer (JEOL, Tokyo, Japan).

Fourier transform infrared (FTIR) spectroscopy: FTIR spectra were measured using the KBr tablet method. The spectral range was 400–4000 cm^−1^, the resolution was 4 cm^−1^, and the accumulative number of scans was 6 times. Each sample was subject to background scanning before analysis. FTIR spectra were baseline corrected to determine the peak value and absorbance. The FTIR analysis was performed with a Vertex 70 spectrometer (Bruker, Billerica, MA, USA).

Anatomical analysis: Cotton petioles with brown rings were cut into 0.2 cm blocks and fixed in FAA (formaldehyde, acetic acid, and ethanol). The samples were evacuated with a SHZ-D (m) circulating water vacuum pump for 40 min, dehydrated with alcohol and xylene to be transparent, and then incubated with shredded wax in a YLD-2000 electric thermostat incubator for at least 4 d. Thereafter, the samples were embedded in paraffin and cut into 6–8-μm-thick sections with a microtome. The section samples were expanded in a KD-H tissue slide warmer and then dried in a DGX-9091B electric thermostat blast oven for 3 d. After safranin O-fast green and toluidine blue staining, the samples were prepared as permanent sections with Canada balsam. The sections were dried in a blast oven before microscopic photography using a BX61 fluorescence microscope (Olympus, Tokyo, Japan) equipped with a digital camera. Six samples were selected for each treatment, and six temporary slices were made for each sample. The resolution of Fig. 5A and B was 1.375 μm, and that for Fig. 5a and b was 0.61 μm.

### Determination of physiological parameters

Lignin^54^. Briefly, a certain amount of the sample (m_0_) was dissolved in ethanol and Soxhlet-extracted to remove the pigment. Next, the samples were hydrolyzed with 72% concentrated sulfuric acid, with the tube placed in water and maintained at 30 °C for 1 hour. Then, the mixture was diluted with water to 4%, and treated in the autoclave at 121 °C for 45 min. The cellulose and hemicellulose were hydrolyzed into monomeric sugars with the two-step acid hydrolysis, followed by filtering the mixture through a funnel by suction. The lignin content was determined by burning the residue. The residue was washed with hot distilled water until the lotion was neutral. Residue and funnel at 105 °C drying, weighing its mass m_1_, ashes at 550 °C, the ash and funnel weight m_2_, the lignin content: m_1_-m_2_/m_0_

Total B was extracted from dry sieved functional leaves with 1 M HCl^55^. Different forms of B were extracted from fresh functional leaves following a three-step procedure^41^. Briefly, frozen leaf samples were thawed and cut into 1 mm^2^ pieces. Approximately 5 g of leaf samples was weighed into 30 mL of redistilled water and oscillated at 25 °C for 24 h, followed by filtering the extracts and collecting the filtrates for determination of free-B. Next, the residuals were transferred into plastic bottles with 1 M NaCl and oscillated at 25 °C for 24 h, followed by filtering the extracts and collecting the filtrates for determination of semi-bound B. Finally, the residuals were transferred into plastic bottles with 1 M HCl and oscillated at 25 °C for 24 h, followed by filtering the extracts and collecting the filtrates for determination of bound B. The concentration of B extracts was measured by inductively coupled plasma mass spectrometry.

Chlorophyll content of fresh functional leaves was measured using a SPAD-502 PLUS meter (Konica Minolta, Japan). Each leaf was measured four times and the mean SPAD value was calculated. Each treatment had six replicates. Photosynthetic rate was measured using a Li-6400XT Photosynthesis System (LI-COR Biosciences, Lincoln, NE, USA), with the photosynthesis-related parameters as follows: PPFD 1200 μmol m^−2^ s^−1^ at the leaf level; temperature 28 °C; relative humidity 60%; CO2 concentration 400 μmol mol^−1^, gas flow rate 300 mol^−1^. Photosynthetic measurements were conducted on the newest fully expanded leaves (the fourth expanded leaf from the top) between 09:00 h and 11:30 h.

### Data analysis

Excel 2013 (Microsoft Corp., Redmond, WA, USA) and SPSS Statistics 17.0 (SPSS Inc., Chicago, IL, USA) were used to draw graphs and run statistical analyses. Multiple comparisons were performed using Duncan’s new multiple range test (*p* < 0.05). Image data were analyzed using Image-Pro Plus 6.0 (Media Cybernetics Inc., Rockville, MD, USA).

### Data availability

All data generated or analysed during this study are included in this published article (and its Supplementary Information files).
